# Handbooks for Medical Students

**Published:** 1906-09-01

**Authors:** 


### HANDBOOKS FOR MEDICAL STUDENTS.

THE text-books of surgery most used by English students are either written by a single author, by two collaborators, or they are collections of articles by different hands, the conflicting statements and the varying experience of the writers being more or less harmonised by an editor. Each group has its advantages and disadvantages. The book written by a single person or by two acting together gives the views from a single standpoint clearly and succinctly. The book is generally of moderate size, is readable, and often has a style of its own. It is well adapted for the use of a student who is going to lay his foundation of knowledge upon a sure basis. The drawbacks, of course, are that no man is equally attached to every branch of surgery or is equally conversant with all its parts. Either he will hate fractures and love abdominal operations, or he will cleave to orthopædics and disdain cleft palates. But an author may successfully hide such idiosyncrasies, and the more successfully if he choose a collaborator with tact and care. Of such text-books the English language is fortunate in possessing some first-rate examples. There are:—

1. "The Manual of Surgery," by Charles Stonham, published by Messrs. Macmillan and Co., in three vols. A thoroughly practical book containing in a readable form and in moderate compass all that a student need know about surgery whilst he is acting as a dresser. And, knowing this, he will afterwards be able to add to and supplement his knowledge by practice and further reading.

2. Walsham's "Theory and Practice of Surgery," edited by W. G. Spencer, published by Messrs. Churchill. The favour in which this book is held is shown by the fact that it has already reached its ninth edition, whilst the editorial care which is bestowed upon it is proved by the increasing size of each edition. The book contains a very large amount of information in comparatively few pages, and for this reason it is a little difficult to read in at all a dilettante fashion. It requires that the student should sit down, work hard and thoroughly master its contents. When he has done so he will begin to know something about surgery.

3. Rose and Carless' "Manual of Surgery," published by Messrs. Baillière and Tindal. This text-book runs neck and neck with the preceding in popular favour and is now in its sixth edition. It has the advantage of being still under the care of the original writers. Its format is perhaps a little more pleasing that that of its rival.

4. Cheyne and Burghard's "Manual of Surgical Treatment" is published in six parts by Messrs. Longmans, Green, and Co. This book is very complete, is thoroughly practical, and is in every way satisfactory for a senior student and for those who wish to know about surgery as it is conducted in England at the present time. Amongst other excellencies it is concerned with the details of surgical treatment. It consists of six good-sized volumes, and is correspondingly expensive.

5. Jacobson and Steward's "Operations of Surgery," published by Messrs. Churchill, in two volumes, has reached its fourth edition, and is perhaps the most satisfactory English text-book of operative surgery extant. It gives the various methods of performing the more common operations, with the reasons for and against any particular method; the literature of the subject, and the deliberate opinion of one who is himself an acknowledged master of surgery, trained by a long experience at Guy's hospital. The book, moreover, is readable. We understand that the fourth edition is out of print, and that the fifth is in course of preparation by Mr. Jackson, with the assistance of Mr. R. P. Rowlands.

Text-books written by various authors under the guidance of a general editor. This form of text-book is less satisfactory for a student than the type just mentioned, because the variety of statements is apt to lead to confusion. Elementary teaching ought to be dogmatic, and it is only when some advance has been made in a science that the pros and cons of debatable points should be offered for consideration. This is better done *viva voce*, by question and answer, than in the baldness of print. But for senior students and for those who know their work articles by different authors are most serviceable. They show how experience differs and may be fallacious, whilst an article written by an author with special knowledge goes more deeply into the subject, and, if pruned judiciously by the editor, is a permanent gain to the general stock of knowledge.

(*a*) "A System of Practical Surgery," edited by Profs. Von Bergmann, Bruns and Mikulicz, translated into English and edited by Drs. Bull, Frost, Flint, and Martin, issued by Messrs. Williams and Norgate in four vols. This system gives a very complete account of the science and art of surgery at the present time. The articles are well translated, and the book is illustrated. It is useful for students offering themselves for the higher surgical examinations, as well as those who wish to attain a deeper knowledge than is desired by the majority of general practitioners.

(*b*) "A System of Surgery," edited by Sir Frederick Treves, in two volumes, published by Messrs. Cassell and Co. The work consists of an excellent series of articles by surgeons attached to the various London hospitals, without any attempt to make it encyclopædic. The articles are concerned with the pathology, clinical manifestations and treatment of the various diseases and injuries that come within the scope of surgery, and the subjects which are of the greater importance are those which have been treated with the greater fullness. This system may be supplemented by Sir Frederick Treves' Manual of Operative Surgery, which is also published by Messrs. Cassell, and in two volumes. This manual concerns itself solely with the practical aspects of treatment by operation, with the technical details of operative surgery and with such part of the surgeon's work as comes within the limits of a handicraft. It differs from Mr. Jacobson's book because it does not deal with the indications for operating. It is written by Sir Frederick Treves himself, and it is, therefore, not only easy, but pleasant to read.

Surgical anatomy and surgical pathology form the backbone of surgery as a science. But too many students are content to learn only so much as is sufficient to enable them to obtain a minimum examination knowledge. There are several good English text-books on surgical anatomy. One of the best is certainly M'Lachlan's "Applied Anatomy: Surgical, Medical and Operative," in two volumes, published at Edinburgh, by Messrs. E. and S. Livingstone. It is clear, well written and well illustrated.

Sheild's "Surgical Anatomy," published by Mr. Young J. Pentland, is a smaller work without illustrations, and is meant to be used with the living model. It cannot be simply read through with any advantage. But if a student will secure a complacent friend and mark out upon him with a pencil or piece of chalk the various facts in each page, he will have arrived at a very respectable knowledge of anatomy in its relation to surgery by the time he has finished the book. Rawling's "Landmarks and Surface Markings of the Human Body," published by H. K. Lewis, is somewhat on the lines of the late Mr. Holden's "Medical and Surgical Landmarks." Mr. Rawling's book is well illustrated, and has already reached a second edition.

Leaf's "Surgical Anatomy of the Lymphatic Glands," published by Messrs. Archibald Constable and Co., is of greater service to the surgeon than the student. It is a praiseworthy study of the exact position of the lymphatic glands, and is especially useful to those who are operating on cases of malignant disease and of tuberculous adenitis.

Sir Frederick Treves' "Surgical Applied Anatomy" has long been a favourite with students. A revised edition has recently been issued by Dr. Arthur Keith, and published by Messrs. Cassell and Co. It is in one volume well illustrated.

Messrs. Sherratt and Hughes, of Manchester, issue a revised edition of "A Handbook of Surgical Anatomy," by Professor G. A. Wright and Dr. C. H. Preston.

Messrs. Box and McAdam Eccles have recently published, through Messrs. Churchill, a book on "Clinical Applied Anatomy," which is in many respects an interesting volume, reminding the reader a little of Hilton's "Rest and Pain," which is so well known to every thoughtful student of surgery. It indicates the important influence of anatomy on the incidence and progress of disease, disorder and injury of the human body.

Surgical pathology can only be learnt in the post-mortem room and the museum. No attempt should be made to learn it by reading. The text-book should be taken into the museum and the description which it contains ought to be verified by the different specimens to be found in every well-arranged collection. The students at St. Bartholomew's Hospital are especially well placed in respect of surgical pathology, for two text-books deal largely with specimens drawn from the St. Bartholomew's Hospital Museum. Mr. Bowlby's "Surgical Pathology" (Messrs. J. and A. Churchill) contains a large number of drawings, and describes in simple language the various pathological processes which are interesting to the student of surgery.

Walsham's "Surgical Pathology" is a catalogue *raisonnée* of the Museum at St. Bartholomew's Hospital. The third edition, lately published by Mr. Paterson, has been adapted for use in pathological collections other than that for which it was originally written.

Buchannan's (A. M.) "Manual of Anatomy" (Baillière, Tindall, and Cox) is in two parts, each 12s. 6d., and forms an excellent work for ordinary students or for honours examinations. Its accuracy and fullness are only equalled by its lucidity of expression and admirable arrangement.

### Special Subjects.

Messrs. Ashby and Wright's "Diseases of Children" (Messrs. Longmans, Green and Co.) is convenient because it contains the medical and surgical diseases in a single volume.

Mr. Edmund Owen's "Surgical Diseases of Children" (Messrs. Cassell and Co.) and Mr. D'Arcy Power's "Surgical Diseases of Children and their Treatment by Modern Methods" (Mr. H. K. Lewis), are well illustrated, and give the views of experienced surgeons who have been attached for many years to large children's hospitals.

### Orthopædics.

"Deformities: A Treatise on Orthopædic Surgery," by Mr. A. H. Tubby (Messrs. Macmillan and Co.) is certainly the best and most complete text-book by an English surgeon in this department of surgery. It may be supplemented by "The Surgery of Paralyses," by Messrs. Tubby and Robert Jones, which describes in a small compass the modern methods of treating the sequelæ of congenital and acquired forms of paralysis by muscle-grafting, tendon transplantation and arthrodesis.

"Orthopædic Surgery: A Text-book of the Pathology and Treatment of Deformities," by Mr. Jackson Clarke (Messrs. Cassell and Co.) is a successful attempt to give an outline of the various therapeutic measures applicable to deformities, the treatment being based upon the pathology of the condition.

### Diseases of the Throat, Nose and Ear.

"Diseases of the Nose and Throat," by Dr. De Havilland Hall and Mr. Herbert Tilley, fully maintains the reputation of Mr. H. K. Lewis' practical series. It forms an easy introduction to a special and important branch of practice which is too often neglected by students because of the slight initial difficulty of learning to use the laryngoscope.

"Diseases of the Throat, Nose and Ear," by Dr. McBride (Young J. Pentland) conveys within a volume of reasonable dimensions the main facts of modern laryngology, rhinology and otology.

Mr. Mark Hovell's "Treatise on Diseases of the Ear and Naso-Pharynx" (Messrs. J. and A. Churchill) gives a very thorough account of aural surgery, and of the treatment of the affections of the nose and pharynx which conduce to disease of the ear.

Dr. William Lamb's "Guide to the Examination of the Throat, Nose and Ear" (Baillière, Tindall, and Cox, 5s.) is one of the most valuable books of the kind. It is practical and suitable to all workers in that speciality.

Dr. Wyatt Wingrave's "Adenoids" (Baillière, Tindall, and Cox, 2s. 6d.) is an exhaustive and essentially practical account of this condition, sound in views and a safe guide, and can be confidently recommended.

"Diseases of the Upper Respiratory Tract," by Dr. Watson Williams, is a most useful handbook, suitable to the student and the practitioner. Its illustrations are particularly good.

Dr. James B. Ball's "Handbook of the Diseases of the Nose and Pharynx" (Ballière, Tindall, and Cox, 7s. 6d.) devotes special care and attention to the question of treatment.

### THERAPEUTICS AND PHARMACOLOGY.

"Text Book of Pharmacology and Therapeutics." Edited by W. Hale White. Young J. Pentland.

"Handbook of Therapeutics." By Ringer and Sainsbury. H. K. Lewis.

"System of Practical Therapeutics" (4 vols.). By H. Amory Hare and Walter Chrystie. Young J. Pentland.

"Text-book of Medical Treatment." By Nestor Tirard. J. and A. Churchill.

"Dictionary of Treatment." By Sir Wm. Witla. Henry Renshaw.

"Manual of Medical Treatment or Clinical Therapeutics." By I. Burney Yeo (2 vols.). Cassell and Company.

